# Amyloid-beta transporter expression at the blood-CSF barrier is age-dependent

**DOI:** 10.1186/2045-8118-8-21

**Published:** 2011-07-08

**Authors:** Crissey L Pascale, Miles C Miller, Catherine Chiu, Matthew Boylan, Ilias N Caralopoulos, Liliana Gonzalez, Conrad E Johanson, Gerald D Silverberg

**Affiliations:** 1 Warren Alpert Medical School Brown University, RI Hospital Department of Neurosurgery 593 Eddy St. Providence, RI 02903 USA; 2 University of Rhode Island Department of Computer Science and Statistics Kingston, RI 02881 USA

**Keywords:** aging, amyloid-beta, transport, choroid plexus, blood-CSF-barrier, LRP-1, LRP -2, P-gp, RAGE

## Abstract

**Background:**

Age is the major risk factor for many neurodegenerative diseases, including Alzheimer's disease (AD). There is an accumulation of amyloid-beta peptides (Aβ) in both the AD brain and the normal aging brain. Clearance of Aβ from the brain occurs via active transport at the blood-brain barrier (BBB) and blood-cerebrospinal fluid barrier (BCSFB). With increasing age, the expression of the Aβ efflux transporters is decreased and the Aβ influx transporter expression is increased at the BBB, adding to the amyloid burden in the brain. Expression of the Aβ transporters at the choroid plexus (CP) epithelium as a function of aging was the subject of this study.

**Methods:**

This project investigated the changes in expression of the Aβ transporters, the low density lipoprotein receptor-related protein-1 (LRP-1), P-glycoprotein (P-gp), LRP-2 (megalin) and the receptor for advanced glycation end-products (RAGE) at the BCSFB in Brown-Norway/Fischer rats at ages 3, 6, 9, 12, 20, 30 and 36 months, using real time RT-PCR to measure transporter mRNA expression, and immunohistochemistry (IHC) to measure transporter protein in isolated rat CP.

**Results:**

There was an increase in the transcription of the Aβ efflux transporters, LRP-1 and P-gp, no change in RAGE expression and a decrease in LRP-2, the CP epithelium influx transporter, at the BCSFB with aging. Decreased Aβ42 concentration in the CP, as measured by quantitative IHC, was associated with these Aβ transporter alterations.

**Conclusions:**

Age-dependent alterations in the CP Aβ transporters are associated with a decrease in Aβ42 accumulation in the CP, and are reciprocal to the changes seen in these transporters at the BBB, suggesting a possible compensatory role for the BCSFB in Aβ clearance in aging.

## Background

Advancing age is a major risk factor for many neurodegenerative disorders, and the major risk factor for Alzheimer's disease (AD), a disease characterized by progressive memory and cognitive loss [1]. The most accepted hypothesis for the mechanism of brain injury in AD is the "amyloid cascade," comprising amyloid accumulation in the brain, the formation of toxic oligomeric and intermediate forms of amyloid-beta peptides (Aβ), amyloid plaques, inflammation and the induction of neurofibrillary tangles [2-4]. There is accumulation of Aβ in both the normal aging brain and the AD brain, thought to be related to defective Aβ clearance rather than increased Aβ production [4-7]. This has recently been shown to be the case in AD [8]. Clearance of this peptide from the brain occurs via active transport at the interfaces separating the central nervous system from the peripheral circulation.

We have recently shown that the blood-brain barrier (BBB) undergoes significant alterations in Aβ transporter expression with aging [5,9]. These changes likely lead to a decrease in amyloid efflux from the brain and an increase in amyloid influx, associated with an increasing CNS amyloid burden. Similar Aβ transporter expression changes have been shown in AD brains [10-12]. The blood-CSF barrier (BCSFB) also undergoes age-related changes, and it is important to study the effects of aging on this alternative pathway for transporter-mediated amyloid clearance [13,14].

BBB Aβ clearance is an energy-dependent (active) process via specific transporter proteins [15,16]. The receptors for Aβ at the BBB bind Aβ directly, or bind to one of its carrier proteins, and transport it across the endothelial cell. The low density lipoprotein receptor-related protein 1 (LRP-1) and P-glycoprotein (P-gp) have been implicated in Aβ efflux [17,18]. LRP-1 is located on the abluminal endothelial cell membrane, whereas P-gp is located on the luminal (blood-facing) surface. The receptor for advanced glycation end products (RAGE), also located on the luminal side of the endothelium, has been linked to Aβ influx [19].

It has been hypothesized that AD is on a continuum with normal aging, although the exact causes for the transition from aging to AD are not yet fully understood [14,20]. In addition to the reduction in clearance of Aβ across the BBB with increased age, there is also a decline in its clearance via CSF bulk flow [21]. We have also demonstrated that interfering with the bulk flow of CSF by induced hydrocephalus causes amyloid to accumulate in the aged rat brain [22]. An increase in ventricular size and a decrease in CSF production and turnover have been demonstrated in senescence, AD and hydrocephalus of the elderly [21,23,24]. Decreased CSF turnover has been postulated to be an important risk factor for AD [7,14].

Solute transport at the BCSFB occurs across the choroid plexus (CP) epithelium [25,26]. Although CSF bulk flow decreases with age and AD, active transport of solutes, such as Aβ, across the CP may not. The same Aβ transporters located on the endothelial cells of the BBB are also found at the BCSFB, e.g., LRP-1, P-gp and RAGE, as well as another receptor known to transport Aβ from the CSF into the CP epithelium, the low density lipoprotein receptor-related protein 2 (LRP-2). Although the LRP receptors may transport solutes bi-directionally, there is evidence that LRP-1 and P-gp are Aβ efflux transporters and LRP-2 primarily transports Aβ from CSF into the CP epithelium [27-30]. Herein, we report the changes in mRNA expression and receptor protein density of these Aβ transporters on the CP epithelium as a function of age. Expression of receptor mRNA was measured by real time reverse transcription-polymerase chain reaction (RT-PCR). Immunohistochemistry (IHC) was used for protein sub-cellular localization and semi-quantitative measurements of receptor expression. IHC was also used to measure Aβ40 and Aβ42 accumulation in CP with advancing age. Interestingly, the overall changes in expression that we found at the BCSFB were reciprocal to those seen at the BBB, and different from AD CP.

## Methods

### Tissue collection

All experiments were approved by the Rhode Island Hospital Institutional animal care and use committee. Male Brown-Norway/Fischer (B-N/F) rats (n = 254) were purchased from the National Institute on Aging at ages 3, 6, 9, 12, 20, 30, and 36 mo. B-N/F rats are not as susceptible to cancer as the more inbred species and can live in excess of 36 mo. Under a surgical plane of anesthesia, induced by intraperitoneal injection of pentobarbital (50 mg/kg), the animals were perfused with 0.9 M phosphate buffered saline (PBS), pH 7.4, at 4°C via the left ventricle of the heart, using a peristaltic pump (Harvard Apparatus, Holliston, MA, USA). The two lateral ventricle CPs were then dissected out under a microscope and placed in a microcentrifuge tube containing either 4% paraformaldehyde (PFA) for fixation prior to IHC, or RNAlater (Ambion, Austin, TX, USA) to limit degradation by endonucleases before RT-PCR analysis. Eight CPs from four rats were pooled for each sample used downstream for PCR, and two CPs from one rat were used for each IHC sample. For PCR, n = 8 (32 rats) were tested for each age group and for IHC n = 5 (five rats) were used for each age group.

### Real time RT-PCR

Samples stored in RNAlater were allowed to thaw and were rinsed with nuclease-free water before homogenizing in the lysis buffer provided in the Qiagen RNeasy kit (Qiagen, Valencia, CA, USA). This kit was used, per the manufacturer's instructions, to extract the RNA. RNA concentrations were measured by a NanoDrop *1000 *spectrophotometer (ThermoFisher Scientific, Wilmington, DE, USA) and stored at -80°C until further use. 1 μg of RNA was used with the Omniscript Reverse Transcription kit (Qiagen) to synthesize 20 μL of cDNA. Forward (F) and reverse (R) primers for each gene of interest were designed using Primer Premier software (PREMIER Biosoft International, Palo Alto, CA, USA). The primers used were as follows (written from 5' to 3'): LRP-1 F *CAAGATGTATGAAGGTGGAGAGC*, LRP-1 R A*CTGGGTTGGTGAAGTTGGTAG *(T_A _= 62°); LRP-2 F *GCAGAGATGGACAGTGAGGT, *LRP-2 R *GCTGGCGAGGCTATACG *(T_A _= 62°); P-gp F *GGACAAAGCCAGGGAAGG*, P-gp R *GGTGGGTGCCGTGCTC *(T_A _= 60°); RAGE F *GCAGGCTCTGTGGATGGG*, RAGE R *GAGTCTGGGTTGTCGTTTTCG *(T_A _= 62°); and βACT F *AAAGACCTCTATGCCAACACAGT*, βACT R *GAGCCACCAATCCACACAG *(T_A _= 60°). In each 50 μL real time RT-PCR reaction using SYBR-ER master mix (Invitrogen, Carlsbad, CA, USA), 10 mM primers and 1 μL cDNA were implemented. Reactions were run in a Bio-Rad iCycler (Bio-Rad, Hercules, CA, USA) using Invitrogen's suggested protocol. Annealing temperatures (T_A_) varied and were specific for each primer set. Standards corresponding to each gene were generated by first amplifying template DNA (βACT: rat cerebral cortex; LRP-1 and RAGE: rat lung; LRP-2: rat kidney; P-gp: rat liver) using a set of gene-specific primers in eight 100 μL RT-PCR reactions. An equal volume of phenol:chloroform was then added to the combined product and mixed vigorously for 15 sec. After sitting on ice for 10 min, the mixture was centrifuged for 20 min on maximum speed at 4°C. The aqueous phase was carefully removed to another microcentrifuge tube where 1/10 volume of sodium acetate was added and mixed. An equal volume of 100% ice cold ethanol was added and allowed to sit overnight in a -20°C freezer. The DNA was pelleted, resuspended in 20 μL Tris EDTA buffer, and run on a 2.5% low melting temperature agarose gel (Fisher Scientific, Fairlawn, NJ, USA) in Tris Borate EDTA buffer for 1.5 h. Due to the intercalating dye, Gelred (Biotium, Hayward, CA, USA), that was added to the gel pre-run, the DNA band was visualized under UV light and was then extracted. The DNA was purified from each gel slice using Promega Wizard columns (Promega, Madison, WI, USA). The concentration of DNA was determined using a spectrophotometer (ThermoFisher Scientific) and a serial dilution for each gene of interest was created (10^2 ^to 10^6 ^copies per μL). Standards for a particular gene were run in each reaction corresponding to that gene. Both standards and samples were run in duplicate, and all runs contained an inter-run calibrator to account for any differences between runs. A melt curve was generated for every reaction and viewed to ensure only the gene of interest was being amplified. All data were normalized to the housekeeping gene, β-actin (βACT), before statistical analysis was performed.

### Immunohistochemistry

Specimens stored in PFA were processed, embedded in paraffin, and sectioned at a thickness of 10 μm. After deparaffinization and rehydration, tissue sections were treated with hot (85°C) 10 mM citrate buffer, pH 6, for 20 min. Sections were washed with distilled water and quenched with a peroxidase-blocking reagent (Dako, Carpinteria, CA, USA) for 10 min at room temperature to eliminate endogenous peroxidase activity. After washing in 0.05 M Tris-buffered saline with 0.05% Tween-20 (TBST), pH 7.6, sections were incubated overnight at 4°C with their appropriate primary antibody: rabbit polyclonal to Aβ40 (Linaris, Wertheim-Bettingen, Germany; Cat. # PAK6012, diluted 1:100), rabbit polyclonal to Aβ42 (Linaris, # PAK6023, diluted 1:200), rabbit polyclonal to LRP-1 (Orbigen, San Diego, CA, USA; # PAB-10774, diluted 1:1000), rabbit polyclonal to LRP-2 (Orbigen, # PAB-10775, diluted 1:2000), mouse monoclonal to P-glycoprotein (Abcam, Cambridge, MA, USA; # ab3364, diluted 1:100), or goat polyclonal to RAGE (Abcam, # ab7764, diluted 1:400). After washing the sections in TBST, a horseradish peroxidase (HRP)-labeled polymer conjugated with secondary antibodies: anti-mouse (Dako) or anti-rabbit (Dako), was applied for 30 min at room temperature, in accordance with the EnVision+ System for IHC staining. For RAGE only, the sections were instead subjected to a modified ABC technique using the Vectastain Elite ABC Goat Peroxidase system (Vector Laboratories, Burlingame, CA, USA). All tissue sections were washed in TBST and stained using 3,3-diaminobenzidine (Dako) as the chromogen. Following development, P-gp sections were counterstained for 30 sec with a 1% light green SF yellowish solution (Sigma, St. Louis, MO, USA). Sections were dehydrated through a series of graded alcohols and xylene, and then coverslipped and sealed using Cytoseal XYL, a xylene-based mounting medium (Richard-Allan Scientific, Kalamazoo, MI, USA). Primary antibody omission controls were run alongside the other samples to check for non-specific binding due to the secondary antibody, along with positive control tissue (human hippocampus with Braak and Braak Stage VI AD pathology, human lung, and human kidney).

### Image Analysis

All IHC slides were converted to digital images using Aperio ScanScope (Aperio Technologies, Vista, CA, USA) as 8-bit acquisitions of color. Image analysis was completed in Image J (v. 1.43 u, NIH, Bethesda, MD, USA). For all stains except for P-gp, stained tissue was selected on the Red Channel based on a specific threshold value, and the 'Mean Gray Value' was measured from the selection. This value renders the average stain intensity as grayscale units (GU) for all thresholded pixels. Quantification of P-gp immunoreactivity was determined by the pixel ratio of stained tissue area to total tissue area [% region of interest (ROI)]. IHC images were taken for six age groups: 3, 6, 12, 20, 30, and 36 mo. Our semi-quantitative IHC methods have been shown to correlate very closely with western blot measurements of Aβ receptor protein at the BBB [5,9].

### Statistical methods

For the RT-PCR analysis, Bio-Rad iQ5 Optical System software (Bio-Rad, Hercules, CA, USA) using the ΔΔC_T _method to calculate the normalized expression of each gene for each sample was employed. Natural logarithm transformations of the data were used to correct for non-normality and inequality of variances, which was then confirmed by Shapiro-Wilk's and Levene's tests. For both the RT-PCR and the IHC analyses a single-factor ANOVA followed by Tukey's pairwise comparisons were used to analyze the data. The confidence limits (CL) for differences in the means (pairwise comparisons) were calculated for the mean of higher - mean of lower age group. All statistical analyses were conducted using the SAS software (V. 9.2, SAS Institute, Cary, NC, USA).

## Results

### LRP-1

**Figure 1:**
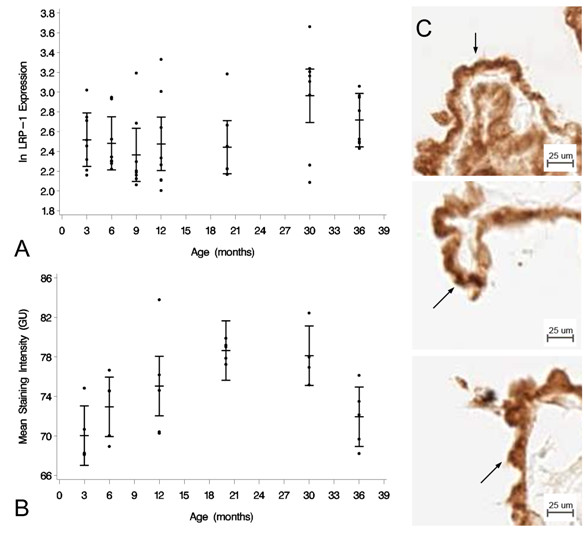
**LRP-1 expression at the CP epithelium with age**. (A) Graph of the log-transformed normalized expression of LRP-1 with age, n = 8 for each age group tested (4 rats, 8 CPs pooled for each "n", 32 rats for an n = 8). One-way ANOVA revealed a significant increase in LRP-1 expression (*p *= 0.047). Significance was reached between 9 and 30 mo 95% confidence limits (0.0122, 1.1828). Error bars represent 95% confidence intervals for the means*. (*B) Semi-quantitative IHC for LRP-1 expression in grayscale units (GU). Mean staining intensity was significantly different (*p <*0.05) for the six age groups, n = 5 for each age group (5 rats, 2 CPs for each sample). (C) IHC of LRP-1 expression at 3 mo (top), 30 mo (center) and 36 mo (bottom) old rats. Staining is localized to the apical membrane (arrows).

The expression of the Aβ efflux transporter LRP-1 at the messenger RNA (mRNA) level as a function of aging was measured by real time quantitative RT-PCR at 3, 6, 9, 12, 20, 30, and 36 mo. There was a mild decrease in LRP-1 expression from 3 to 9 mo, followed by a significant increase to 30 mo and then a mild decrease again to 36 mo (Figure 1A). A one-way ANOVA showed an effect of age for the seven tested age groups at the *p *< 0.05 level [F(6, 49) = 2.33, *p *= 0.047]. Tukey's pairwise comparisons revealed a significant difference in the mean natural log-transformed normalized expression of LRP-1 between 9 and 30 mo [difference between means (DMB) = 0.5975; 95% confidence limits (CL) (0.0122, 1.1828)]. However, the 3, 6, 12, 20, and 36 mo groups did not significantly differ from each other, or from the 9 and 30 mo groups. From 3 to 36 mo, therefore, CP LRP-1 expression was either stable or increased with age.

Semi-quantitative image analysis of the IHC for the LRP-1 protein receptor revealed a similar expression change with aging (Figure 1B). The mean staining intensity was measured in grayscale units (GU). LRP-1 expression was different at the *p *< 0.05 level for the six different age groups [F(5,24) = 5.27, *p *= 0.0021]. Tukey's pairwise comparison showed a significant increase in mean staining intensity (GU) between 3 and 20 mo [DBM = 8.633; 95% CL (2.052, 15.215)] and 3 and 30 mo [DBM = 8.105; 95% CL (1.524, 14.687)]. There was a significant decrease from 20 to 36 mo, [DBM = -6.707, 95% CL (-13.288, -0.125)]. LRP-1 staining appeared to be most intense along the apical membrane of the CP epithelial cell (Figure 1C).

### LRP-2

**Figure 2:**
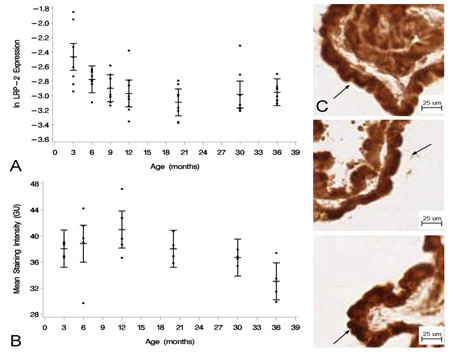
**Expression of LRP-2 at the CP with age**. (A) Graph of the log-transformed normalized expression of LRP-2 with respect to age, n = 8 per age group. One-way ANOVA revealed a significant decrease (*p *= 0.0005) with age on LRP-2 expression. Significance was reached between 3 and 9, 12, 20, 30, and 36 mo. Error bars represent 95% confidence intervals for the means. (B) Semi-quantitative IHC for LRP-2 expression in grayscale units (GU). Mean staining intensity was significantly decreased (*p <*0.05) for the age groups, n = 5 per age group. (C) IHC of LRP-2 expression at 3 mo (top), 20 mo (center), and 36 mo (bottom) old rats. Staining is localized to the apical membrane (arrows), and is also seen subapically.

The expression of the Aβ CP influx transporter LRP-2 at the mRNA level with age was also measured by RT-PCR at the same time points as LRP-1. There was a decrease in LRP-2 expression from 3 to 36 mo (Figure 2A). One-way ANOVA showed an effect of age for the seven age groups at the *p *< 0.05 level [F(6, 49) = 4.93, *p *= 0.0005]. Tukey's pairwise comparisons revealed significant differences in the mean natural log-transformed normalized expression of LRP-2 between 3 and 9 mo [DBM = 0.4323; 95% CL (-0.8340, -0.0305)], 3 and 12 mo [DBM = 0.5061; 95% CL (-0.9078, -0.1044)], 3 and 20 mo [DBM = 0.6270; 95% CL (-1.0287, -0.2253)], 3 and 30 mo [DBM = 0.5183; 95% CL (-0.9201, -0.1166)] and 3 and 36 mo [DBM = 0.4887; 95% CL (-0.8904,-0.0869)]. The results show a continuous decrease in the expression of LRP-2 at the CP epithelium after 3 mo of age.

Semi-quantitative image analysis of the IHC for the LRP-2 protein receptor showed similar results (Figure 2B). The mean staining intensity was different at the *p *< 0.05 level for the different age groups [F(5,24) = 3.47, *p *= 0.0168]. Tukey's pairwise comparison showed a significant decrease in mean staining intensity between 12 and 36 mo [DBM = -7.924; 95% CL (-14.199, -1.729)]. There also appeared to be some localization of the LRP-2 staining to the apical membrane of the CP epithelium, but subapical staining was also evident (Figure 2C).

### P-gp

**Figure 3:**
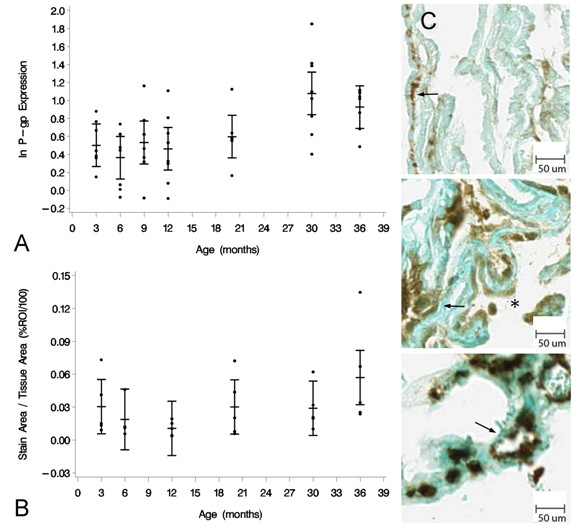
**Age-related P-gp expression at the CP**. (A) Graph of the log-transformed normalized expression of P-gp with respect to age, n = 8 for each age group. One-way ANOVA revealed a significant increase (*p* = 0.0005) with age on P-gp expression. Significance was reached between 30 mo and 3, 6, 9, and 12 mo, and between 36 mo and 6 mo. Error bars represent 95% confidence intervals for the means. (B) Semi-quantitative IHC for P-gp expression. Stain area to tissue area ratio was not significantly different (p > 0.05) for the six age groups, n = 5 per age group. (C) IHC of P-gp expression at 3 mo (top), 20 mo (center), and 36 mo (bottom) old rats. Majority of staining is localized to the basolateral membrane (arrows), though on occasion it is seen along the apical membrane (asterisk).

The expression of P-gp as a function of age, measured by RT-PCR, showed an increase from 3 to 36 mo (Figure 3A). A one-way ANOVA showed an effect of age for the seven age groups at the *p <*0.05 level [F(6, 49) = 4.90, *p *= 0.0005]. Tukey's pairwise comparisons indicated a significant increase in the mean natural log-transformed normalized expression between 3 and 30 mo [DBM = 0.5759; 95% CL (0.0603, 1.0915)], 6 and 30 mo [DBM = 0.7134; 95% CL (0.1978, 1.2290)], 9 and 30 months [DBM = 0.5454; 95% CL (0.0298, 1.0610)], 12 and 30 mo [DBM = 0.6153; 95% CL (0.0997, 1.1309)], and between 6 and 36 mo [DBM = 0.5620; 95% CL (0.0464, 1.0776)]. These results indicate that P-gp expression in the CP epithelium increases with age at the mRNA level. This effect becomes significant at 30 mo. It should be noted that the increase at 36 mo was only significant when compared to the 6 mo age group.

Due to the punctate nature of the P-gp IHC staining, semi-quantitative image analysis of the IHC for the expression of P-gp (Figure 3B) compared the ratio of stained tissue area (pixels) to total tissue area (pixels) rather than mean stain intensity. No significant difference in this ratio was found for the different age groups at the *p *< 0.05 level, though there was a general trend towards increased expression with age. P-gp staining was mainly localized to the basolateral CP epithelial membrane, though it was occasionally seen at the apical membrane as well (Figure 3C).

### RAGE

The expression of RAGE with respect to age was also measured by RT-PCR. One-way ANOVA assessed the effect of age on the mean natural log-transformed normalized expression of RAGE at 3, 6, 9, 12, 20, 30 and 36 mo. There was no significant effect of age on the expression of RAGE at the *p <*0.05 level [F(6, 49) = 0.88, *p *= 0.52]. Image analysis for the IHC performed for the RAGE protein confirmed that no significant differences between any of the age groups were present (*p *= 0.4041). RAGE staining did not appear to localize to CP membranes but rather was cytosolic (PCR and IHC data not shown).

### Aβ40/42

**Figure 4:**
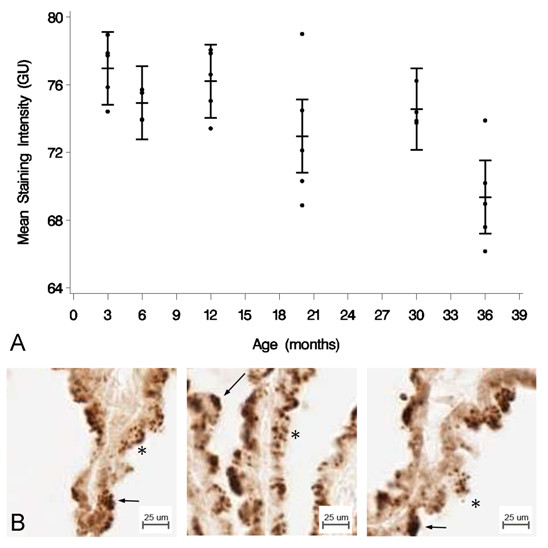
**Aβ42 concentration in the CP epithelium with age**. (A) Semi-quantitative IHC for Aβ42 deposition. Mean staining intensity in grayscale units (GU) was significantly decreased (*p *< 0.05) for the age groups, n = 5 per age group. (B) IHC of Aβ42 at 3 mo (left), 20 mo (center), and 36 mo (right) old rats. Staining is granular and primarily cytosolic (asterisks), though also found along the apical membrane (arrows).

IHC for Aβ40 and Aβ42 deposition was also analyzed semi-quantitatively. The mean staining intensity was not significantly different at the *p *< 0.05 level for Aβ40, *p *= 0.3384, (data not shown), but there was a significant decrease with age [F(5, 23) = 6.40, *p *= 0.0007] for Aβ42 (Figure 4A). Tukey's pairwise comparison showed a significant decrease in mean staining intensity for Aβ42 between 3 and 36 mo [DBM = -7.606; 95% CL (-12.337, -2.874)], 6 and 36 mo [DBM = -5.567; 95% CL (-10.299, -0.835)], 12 and 36 mo [DBM = -6.839; 95% CL (11.570, -2.170)], and 30 and 36 mo [DBM = -5.200; 95% CL (-10.219, -0.812)]. Age decreases Aβ42 deposition in the CP epithelium of the B-N/F rat. Aβ42 staining is granular and primarily cytosolic, though it was also found along the apical CP membrane (Figure 4B).

**Figure 5:**
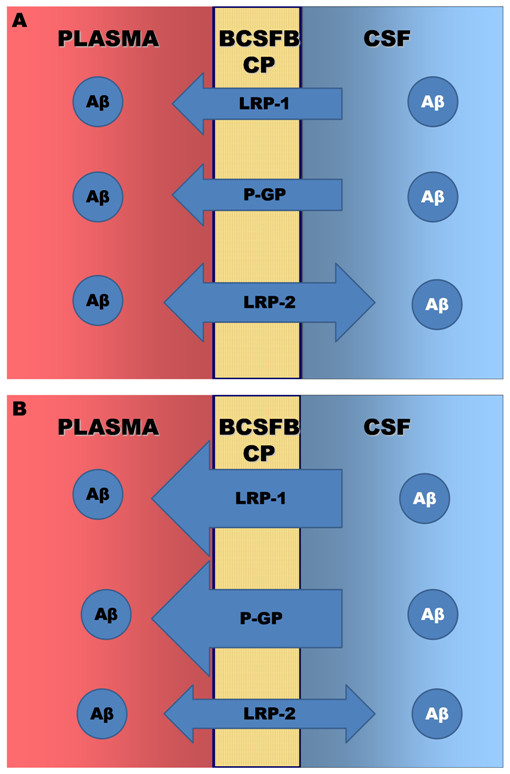
**Diagrams of the direction and expression of the Aβ transporters at the BCSFB**. (A) Diagram to show the direction and relative expression of LRP-1, P-gp and LRP-2 at three months of age in the BN/F rat. (B) Diagram of the relative expression of the three transporters after 20 months. Note that LRP-1 and P-gp expression increase, whereas LRP-2 expression decreases with age.

Figure 5 summarizes the CP Aβ transporters, as we believe they function from the data presented. The figure compares qualitatively the direction and expression of the transporters at three months (A) and after 20 months (B).

## Discussion

Increased amyloid burden in the AD brain is neurotoxic and leads to neuritic plaque formation, chronic inflammation and neuronal death [2,31]. Amyloid accumulation is related to a decrease in Aβ clearance [4,7,8,20]. It has been postulated that the lack of clearance from the brain via transporters at the BBB is the major mechanism behind Aβ buildup in senescence and in AD [5,9,14,16]. We now show that Aβ clearance at the BCSFB may be enhanced with age, reciprocal to Aβ transporter alterations at the BBB.

As noted, most Aβ clearance pathways become compromised in the aged brain. Aside from the alterations in BBB transporter expression, and the attenuation of CSF bulk flow, enzymatic degradation of Aβ by insulin-degrading enzyme and/or neprilysin may also be diminished [32]. The CP epithelial cell surface area is enlarged by the basal labyrinth interdigitations and the apical microvilli, but is still less than the BBB endothelium. However, the contribution to Aβ clearance by the CP is not insubstantial, owing in part to the high levels of Aβ degrading enzymes present [33]. Indeed, with the clearance deficits that occur at the BBB, and the decrease in CSF turnover that occurs with age, the role of Aβ transport and degradation at the BCSFB becomes increasingly more prominent. Between nine and 12 months of age in the B-N/F rat aging model, whole brain Aβ rises rapidly [5]. At 12 months of age CP Aβ42 begins to decrease as P-gp expression starts to increase and LRP-2 expression is decreasing. LRP-1 expression remains stable until 20 months and then increases.

Central to the functioning of the BCSFB is the CP, the organ responsible for monitoring both plasma and CSF concentrations of many solutes, e.g., vitamins, hormones, harmful metabolites and immune cells, and regulating solute concentrations [26,34,35]. The CP likely influences neurogenesis in the damaged brain through the CSF transport of mitogenic growth factors to neurogenic niches near the ventricular wall [36]. In aging and in AD there is a decrease in the production and turnover of CSF allowing toxic metabolites, such as Aβ, more retention time in the brain [7,21,23]. At the same time there is a flattening of the CP epithelium, thickening of the basement membrane, and overall atrophy of the CP cells [37,38]. Therefore, it is of major interest that, despite these dystrophic CP changes, our aging rat CP data shows an increase in expression of the Aβ efflux transporters LRP-1 and P-gp, and either no change (RAGE) or a decrease in the expression of the CP influx transporter (LRP-2) in association with a significant decrease in CP Aβ42 concentration, as measured by IHC. This observation may have a significant bearing on the transition that occurs between normal aging and AD.

Aβ concentration is increased in the CP of AD brains, and the expression of the CP Aβ transporters is altered to decrease Aβ efflux and/or degradation [39,40]. Aβ is shuttled bi-directionally across the BCSFB, and the net flux and rate of Aβ transport is determined by the combined expression and activities of the CP Aβ transporters [41]. The amount of data available at present for the cell membrane localization of the amyloid transporters, and the direction and activity of transport is small. Hence much more work needs to be done in these areas. However, the Aβ efflux transporter expression increase and the decrease in CP Aβ42 suggest a net increase in Aβ efflux at the BCSFB in the aging rat. Loss of this Aβ clearance route may be a determining factor in the increase in CP Aβ seen in AD. Aβ accumulation in the CP increases apoptosis and interferes with oxidative phosphorylation, disrupting the BCSFB and likely further decreasing CSF turnover [40]. Aβ therefore contributes progressively to an inability to clear itself.

The low density lipoprotein receptor (LDLR) family is an ancient and highly conserved family of receptors with endocytic and cell signaling functions. These receptors bind a wide range of ligands, including Aβ [42,43], and LRP-1 has been linked to AD [18,44,45]. LRP-1 has been localized to neurons, glial cells, the endothelial cells of the BBB and the epithelial cells of the BCSFB. It has been shown to bind both Aβ40 and Aβ42, alone, or conjugated to one of their carrier proteins, and remove them from the extracellular space. Once bound, LRP-1 can target Aβ for cellular degradation, or facilitate its transcytosis [46]. A fully functional soluble form, sLRP-1, can also be found in the plasma, brain ISF and CSF [47]. At the BBB, LRP-1 transports Aβ out of the brain. BBB LRP-1 expression has been shown to decrease significantly in aging and in AD [9,10]. At the BCSFB, LRP-1 actively transports Aβ out of the CSF [27].

The present study demonstrates that the mRNA expression and protein levels of LRP-1 increase in CP, and an increase in sLRP-1 in the CSF with advancing age has been reported [47]. It has been shown that Aβ42 can increase the CSF shedding of membrane-bound LRP-1 as sLRP-1 [47]. An increase of sLRP-1 would create an Aβ sink in the CSF, allowing Aβ to accumulate in the CSF by binding to sLRP-1. When bound, Aβ would be inhibited from self-aggregation. The bound Aβ would be transferred to the systemic circulation for degradation.

LRP-2 is another member of the LDLR family known to transport Aβ at the BCSFB. LRP-2 is expressed on capillary endothelium and CP epithelium, though its expression is 17-fold higher in CP epithelium [48]. It has been demonstrated that LRP-2 binds to Aβ40 when it is conjugated to apolipoprotein J (ApoJ) and transports it into the CP epithelial cell from the CSF [29]. LRP-2 also transports solutes from blood into the CP epithelium, and from there into the CSF and brain. LRP-2 has not only been implicated in Aβ transport, but also the transport of leptin and insulin-like growth factor 1 (IGF-1) from the peripheral circulation, across the CP, and into the brain [49,50]. Transport of Aβ into the CP cells may keep it from forming aggregates within the cell, or it could facilitate its degradation by endolysosomes. At the BBB, LRP-2 acts in a manner similar to RAGE, transporting Aβ out of the blood and into brain. However its high saturability with ApoJ not bound to Aβ may restrict its role in Aβ influx transport at the BBB [51]. LRP-2 actions at the BCSFB are less clear. It appears to transport solutes into the CP epithelium from both blood and CSF, and either degrades them within the cell or transports certain ones into the brain via the CSF. The present study finds the transcription of LRP-2 in CP to be significantly down-regulated with age, and its protein levels also decreased. Other groups have shown a similar decrease in the protein levels of LRP-2 at the BCSFB with age and AD [39,49,50]. The decline of LRP-2 expression with age might restrict the passage of Aβ into the CP epithelium; however, this could also restrict certain neuroprotectants like IGF-1 transported by LRP-2 into the CSF and brain. It has been suggested that this lack of neuroprotectant transport leads to cognitive decline [49].

P-gp is a member of the ATP binding cassette superfamily. This is another highly promiscuous receptor which exhibits a high degree of cross-reactivity with a number of unrelated substances. As an efflux transporter it acts to protect the brain at the BBB by pumping xenobiotics out of the brain [28,52]. It has been demonstrated that P-gp is decreased at the BBB late in aging [9,17]. Its role at the BCSFB, however, is poorly understood. In this study, P-gp transcript expression significantly increased with advancing age. IHC localized P-gp primarily to the basolateral membrane of the epithelial cells, suggesting solute transport from the CP into the peripheral circulation. Other studies have not shown this basolateral localization of P-gp. Rao *et al*. (1999) concluded that P-gp was located sub-apically from staining patterns and functional tests [53]. Age seems to be the most likely explanation for this discrepancy between studies. In that study, neonatal CPs and cell cultures were used, rather than adult and aged rats as in the present study. It is possible that cell signaling directs P-gp to different membrane sites at various stages of life.

Another endocytic receptor and transporter of Aβ is RAGE. It is a member of the immunoglobulin superfamily and its ligands include many diverse proteins which have undergone post-translational modifications [54]. As part of the innate immunity of the brain, the interaction between RAGE and its ligands can stimulate an inflammatory response and lead to oxidative stress within cells [55]. RAGE-Aβ interaction at the BBB has also been shown to lead to T-cell infiltration of the brain [56]. At the BBB, RAGE expression increases with advancing age and AD, increasing Aβ influx [5,12]. Few studies have explored the role of RAGE at the BCSFB. One study showed that RAGE increases in the CP after diabetic ketoacidosis, interestingly, without an increase in amyloid [30]. Both the transcript expression and the protein analysis in our study did not show any significant change with increased age, nor did the IHC demonstrate clearly the sub-cellular location of RAGE. It is unclear at this point what the role of RAGE is at the BCSFB in relation to Aβ transport.

Our data show that the Aβ efflux transporter up-regulation at the BCSFB occurs in a manner opposite to that in the BBB, suggesting a possible compensatory role for the CP in eliminating amyloid from the CSF, CP and brain in aging. Although more confirmatory studies need to be done to be certain of the localization and direction of transport of the LRP receptors on the CP, it is important to note that A*β*42 was shown to significantly decrease in the CP with increased age as one would predict from the transporter expressions and directions that we have reported. This differs from the findings in the CPs of AD patients [39,40]. Other studies have also found differences between aging and AD in the CSF levels of sLRPs [47] and transthyretin, known to bind Aβ in the CSF and impede amyloid fibrillogenesis [57]. Both of these proteins are decreased in AD compared to age-matched controls. The changes in Aβ transporter expression at the BBB between aging and AD are in the same direction, though more severe in AD. The differences between aging and AD at the CP and BCSFB may be where the normal aging brain and AD brain differ the most. These differences, and the signaling mechanisms behind them, may provide some insight into the complex relationship between aging and AD.

## Conclusions

Aging alters the expression of the amyloid transporter genes at the BCSFB in a way that may facilitate amyloid clearance across the CP epithelium, i.e., there is an increase in the expression of the Aβ efflux transporters LRP-1 and P-gp, whereas the CP Aβ influx transporter LRP-2 is decreased. The changes in expression of the Aβ transporter genes at the BCSFB are opposite to those seen at the BBB in aging, and may be a compensatory mechanism to aid the failing BBB Aβ clearance pathways. Eventual failure of this compensatory mechanism may have implications for Aβ accumulation in both normal aging and in AD.

## Abbreviations

Aβ: amyloid-beta peptide; Aβ40: 40 amino acid Aβ; Aβ42: 42 amino acid Aβ; AD: Alzheimer's disease; ANOVA: analysis of variance; BBB: blood-brain barrier; BCSFB: blood-CSF barrier; B-N/F: Brown-Norway/Fischer (rat); cDNA: complementary deoxyribonucleic acid; CL: confidence limits; CP: choroid plexus; CSF: cerebrospinal fluid; GU: grayscale units; HRP: horseradish peroxidase; IHC: immunohistochemistry; LRP-1: low density lipoprotein receptor-related protein-1; LRP-2: low density lipoprotein receptor-related protein-2 (megalin); mRNA: messenger ribonucleic acid; PBS: phosphate-buffered saline; PFA: paraformaldehyde; P-gp: P-glycoprotein; RAGE: receptor for advanced glycation end-products; RT-PCR: real time (quantitative) polymerase chain reaction; sLRP-1: soluble LRP-1; TBST: Tris-buffered saline with Tween-20.

## Competing interest disclosures

There are no actual or potential competing interests (conflicts of interest) to disclose for any of the authors or their families.

## Authors' contributions

CP and CC did the real time RT-PCR measurements and CP wrote the first draft of the manuscript. MCM and MB did the immunostaining and edited the manuscript, LG did the statistical analysis, and GDS conceived the research plan, organized the results and edited the manuscript. INC and CEJ reviewed the results and edited and corrected the manuscript. All authors have read and approved the final version of the manuscript.
